# Dynamic interactions between oil price and exchange rate

**DOI:** 10.1371/journal.pone.0237172

**Published:** 2020-08-20

**Authors:** César Castro, Rebeca Jiménez-Rodríguez

**Affiliations:** 1 Department of Economics, Universidad Pública de Navarra, Pamplona, Spain; 2 Department of Economics, Universidad de Salamanca, Salamanca, Spain; Universitat Jaume I, SPAIN

## Abstract

This paper contributes to better understand the dynamic interactions between effective exchange rate (EER) and oil price for an oil-importing country like the U.S. by considering a Time-Varying Parameter VAR model with the use of monthly data from 1974:01 to 2019:07. Our findings show a depreciation after an oil price shock in the short-run for any period of time, although the pattern of long-run responses of U.S. EER is diverse across time periods, with an appreciation being observed before the mid-2000s and after the mid-2010s, and a depreciation between both periods. This diversity of response should lead policy makers to react differently in order to counteract such shocks. Furthermore, the reaction of oil price to an appreciation of U.S. EER is negative and different over time, which may generate different adverse effects on investment. The knowledge of such effects may help financial investors to diversify their investments in order to optimize the risk-return profile of their portfolios.

## 1 Introduction

**Fig 1:**
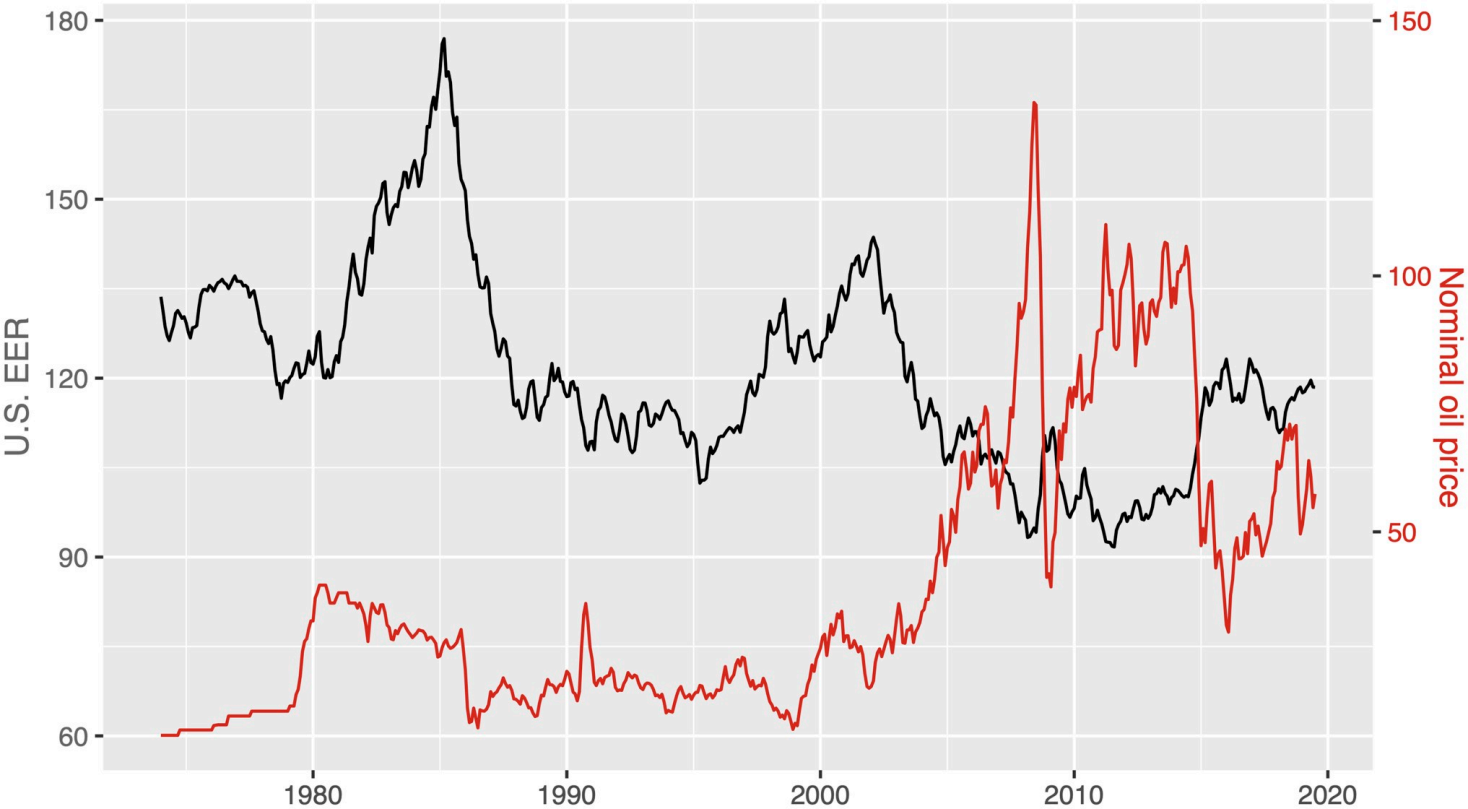
U.S. EER and nominal oil price (1974:01-2019:07). U.S. EER is the U.S. nominal effective exchange rate and the nominal oil price is the spot price of West Texas Intermediate in USD per barrel. Source: Federal Reserve Economic Data and Bank of International Settlements.

**Fig 2:**
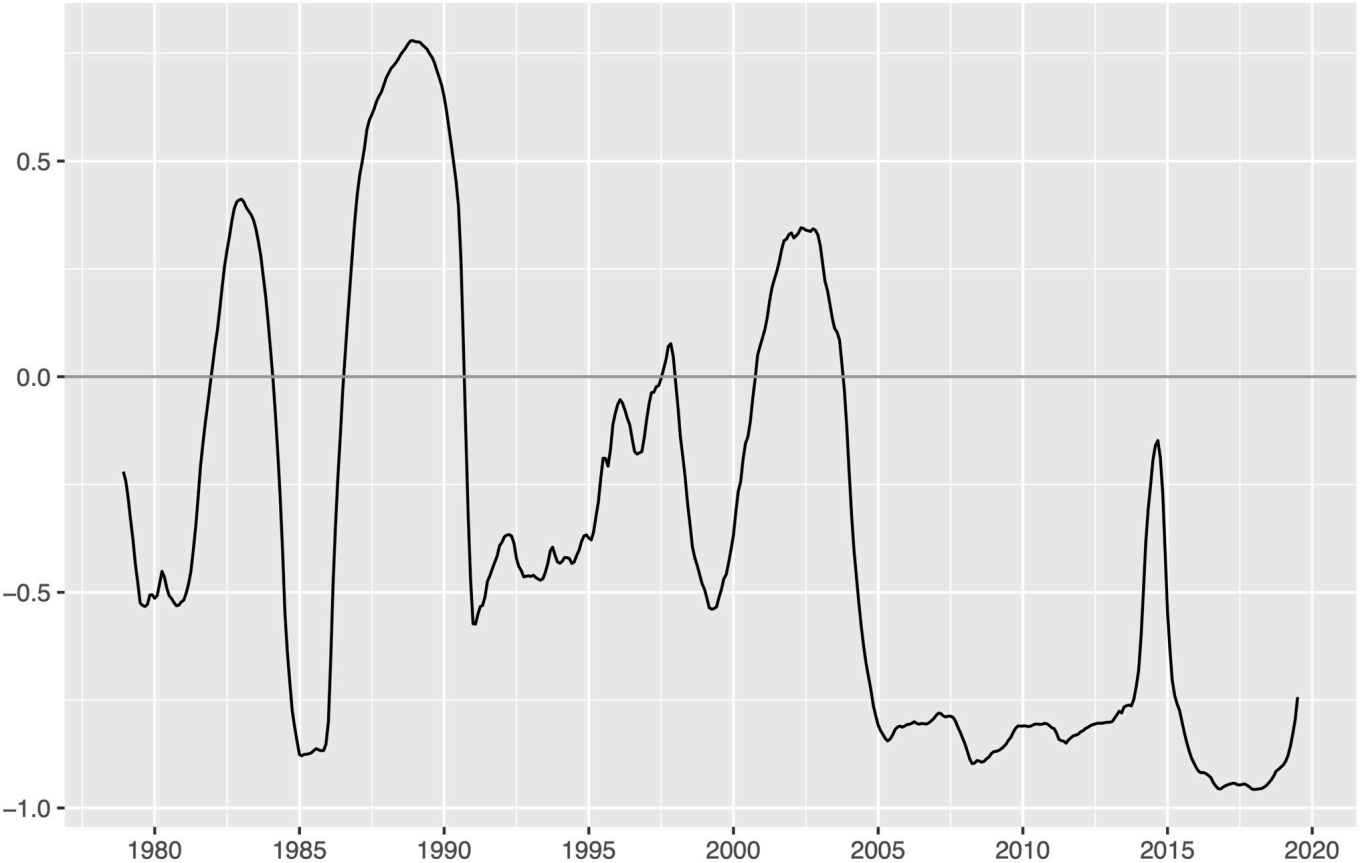
Five-year rolling correlations between U.S. EER and nominal oil price.

The relationship between nominal oil price and U.S. effective exchange rate (EER) seems not to be the same over time. There have been some periods in which both variables have moved in the same direction and other periods in which they have moved in opposite directions (see Fig 1). However, when the correlation between the two variables is calculated for the whole sample, it is simply observed a negative correlation (-0.6), ignoring the varying link between the two variables. Thus, it seems useful to calculate the rolling correlations. To sake of space, we only present and comment the five-year rolling correlations in Fig 2. These correlations provide compelling evidence to support shifts in both the magnitude and the sign (similar instability is found when different sizes of rolling windows are considered). Other authors such as [1] has also found instabilities, showing a very weak correlation between U.S. EER and WTI crude oil price before the early 2000s, but a rising negative correlation since that date reaching the highest value (around -0.6) in the first months of 2009 and 2010. On the one hand, the five-year rolling correlations shown in Fig 2 indicate that the correlation was weak during the period before mid-1980, the nineties and very beginning of the 2000s, but it was strong (higher than 0.6, in absolute terms) for the rest of the periods (except for mid-2014). Thus, whereas the hedge strategy to diversify investment used by financial investors does not fulfill its objective of achieving risk diversification when the correlation is weak, this strategy is effective when the correlation is strong (see, for instance, [2]). On the other hand, we observe that the positive values of the correlation predominate the negative ones in two periods (which coincide with those in which the U.S. dollar -USD- takes particularly high values): the one before September 1990 and that between November 2000 and October 2003, with the average correlation being 0.023 and 0.236, respectively. The first period was characterized by the troubles in world oil market supply in the 1970s (Yom Kippur War, Iranian Revolution and Iran-Iraq War), the aggressive U.S. monetary policy under Paul Volcker and a subsequent relative stable oil price disrupted by the sharp drop in the mid-1980s and the Gulf War in August 1990. The second period was associated to the upward trend caused by global demand in Asia and the remarkable role of oil and other raw commodities as alternative financial assets. Also, Fig 2 displays two periods with basically negative correlations: the one between October 1990 and October 2000 and that from November 2003 onwards (with the average correlation being -0.311 and -0.785, respectively). The first period was characterized by an increase in the Iraqi production and world oil inventories because of warm winters, and a reduction of oil demand due to the Asian crisis. The second period was related to the rise of oil demand from Asia, the sharpest drop caused by the global financial crisis in 2008, the subsequent weak global oil demand and the considerable role played by a larger global supply.

Therefore, the relationship between oil price and U.S. exchange rate seems not to be the same over time and it is needed to use a flexible modelling in order to capture the time varying behavior. To the best of our knowledge, only two recent studies ([3] and [4]) together with the working paper version of this article [5] have analyzed the time-varying relationship between oil price and U.S. exchange rate. In doing so, these studies use time-varying parameter (TVP) VAR models. It is worth noting that TVP-VAR models have been used in some analyses to study the changing impact of oil shocks on U.S. economic growth [6], U.S. stock market [7], or U.S. industrial production [8], as well as in the study performed by [9] to analyze the contemporaneous elasticities of oil price with respect to U.S. dollar, HWWI index (industrial raw materials index), gold price and dry cargo index. Moreover, TVP-VAR models have been widely used to analyze the dynamics of macroeconomic variables not related to oil price (see, e.g., [10]; [11]).

Regarding the studies analyzing the time-varying relationship between oil price and U.S. exchange rate, [3], on the one hand, consider weekly data from 7 January 2000 to 25 July 2014 to study the linear and non-linear causality between the two variables and the effects of structural breaks in the volatility of crude oil and exchange rate markets, but they also dedicate one section of their paper to analyze the time-varying influence of the two variables. Thus, they display the one-period-ahead impulse responses of oil price to an exchange rate shock and those of exchange rate to an oil price shock (without credible intervals) over time in their Fig 2, showing that an appreciation of USD reduces oil price while an increase in oil price has led to a USD depreciation except for the periods 2002-2004 and 2009-2013, where an appreciation is observed. They also show the impulse responses for three specific dates (1 January 2002, 11 July 2008 and 1 January 2010) at different time periods ahead (without credible intervals) in their Fig 3, showing that the impact of exchange rate shocks on oil price is more intense than the effect of oil price shocks on USD, although the latter is more “long-lasting” (specifically, four periods versus one period). Thus, apart from the three specific dates mentioned above, they do not show the responses of each variable to a shock in the other for horizons different from one-period-ahead, but knowing how these responses beyond one-week-ahead is relevant to policy-makers and investors. On the other hand, [4] use a three-variable TVP-VAR model (oil price, U.S. exchange rate and U.S. economic policy uncertainty index) with monthly data from January 1996 to April 2019. Although these authors show both the impulse responses of each variable to a shock in the others (without credible intervals) at three different period horizons (three-, six- and twelve-period horizons) in their Fig 2 and those for the six specific dates (August 2004, August 2007, October 2008, September 2010, November 2014 and November 2018) in their Figs 4 and 5, they only analyze the reaction of exchange rate to shocks in oil price and economic policy uncertainty, as well as the reaction of economic policy uncertainty to oil price shocks. Thus, they ignore the analysis of the effect of shocks in the exchange rate on oil price. Finally, it is worth mentioning that none of these two studies [3, 4] specifies whether the impulse-response functions are referred to responses to either one standard deviation shock or one unit shock. This appraisal is relevant because the standard deviations are considered to be time varying in the specifications of model used.

**Fig 3:**
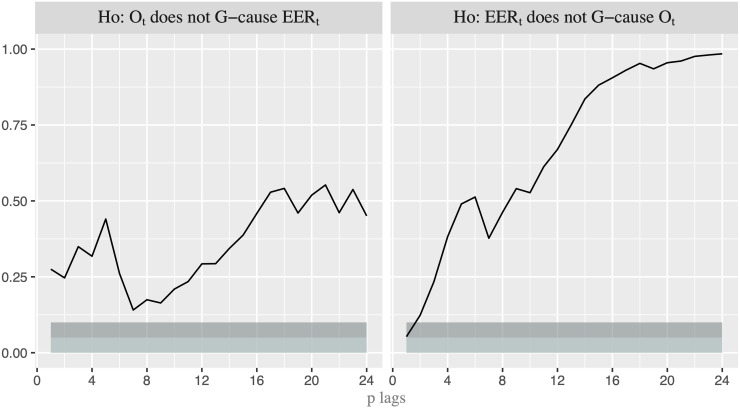
*p-* values for the linear Granger-causality test. p-value for the G-causality test with lags p = 1,…,24. Light shaded area represents the rejection of the null hypothesis at the 5% critical level, while dark shaded area represents the rejection at the 10% level.

This paper considers a recursively identified bivariate TVP-VAR model to analyze the relationship between U.S. EER and oil price from the mid-1970s onwards. As [12] pointed out, a recursively identified bivariate VAR model shows advantages over higher-dimensional VAR models. In particular, the use of large-dimensional VAR models requires additional identifying assumptions that may not be realistic and they tend to be less precisely estimated. In addition, including some extra variables is not required if the objective (as it is in this case) is merely to estimate consistently the response of one variable to the shock of the other. Unlike [3], we analyze the reactions of each variable to a shock in the other for horizons different from one-period-ahead for the whole sample period (not only for specific dates), incorporate credible intervals and analyze how responses are in the period before the 2000s (period in which half of the oil price and exchange rate shocks have occurred). Unlike [4], we also study the response of oil price to shocks in the exchange rate, include the credible intervals and analyze what has happened in the period before the mid-1990s (period in which the most well-known oil price shocks have occurred).

Therefore, it is our aim to study the varying reaction of one variable to the shock of the other in the United States to fill out the gap existing in the recent literature on the analysis of the relationship between oil price and U.S. exchange rate. Our findings will contribute to clarify how the responses of each variable considered to a shock in the other may change over time and how these responses are at different horizons (one or more periods after the shock), as well as the significance of such responses. Notice that the policy-makers’ knowledge of the existence of a diversity of responses of U.S. exchange rate to oil price shock may allow them to react differently to such shocks in order to counteract them. Likewise, the information regarding the effects generated on investment by the reaction of oil price to exchange rate is valuable for financial investors since it may help them to diversify their investment to optimize the risk return profile of their portfolios.

The remainder of the paper is organized as follows. Section 2 introduces a selected literature review. Section 3 describes data. Section 4 presents the methodology. Section 5 displays the results. Section 6 presents some concluding remarks.

## 2 Literature review

The literature on the relationship between oil price and U.S. exchange rate has focused on analyzing the sign and the direction of the causality, but there is no consensus on any of them.

Regarding the direction of causality, some authors [13–15] emphasize the role of the U.S. exchange rate anticipating the movements in oil price, while others [16–18] focus on the reverse anticipation (i.e., oil price changes anticipate movements in U.S. exchange rate). In addition, there are also authors that show the existence of causality in both directions (e.g., [1, 3, 19]). It is worth mentioning that [19] find that a unidirectional causality running from petroleum price to exchange rate in the period before the great crisis, and a bidirectional one afterwards.

The lack of consensus on the direction of causality between oil price and exchange rate not only happens in the U.S., but also with other countries. Thus, the differences in the direction of the causality in the empirical evidence can be related, among others, to three key issues: i) data frequency (for example, in a study for some small exporting countries, [18] argue that commodity prices -including oil price- contain significant valuable information for predicting exchange rate at daily data, while the predictive content is weaker at monthly and quarterly frequency); ii) oil-dependence of the country for each specific period of time (for instance, [18] point out the improvement in the prediction of exchange rate by means of a forecast model including oil price after Canada became a net oil-exporting country); and iii) period of analysis (for example, [15] find a negative relationship between real oil price and U.S. real effective exchange rate when they use the monthly full sample 1974-2015, but this relationship turns positive when the sample ends in the mid-2000s). The latter two issues may have to do with the possible existence of structural breaks, but there is not a clear conclusion about such an existence in the related literature. Thus, [20] do not find evidence of structural breaks for G-7 countries in the relationship between oil price and real exchange rate by using monthly data from January 1972 to October 2005. However, [1] show evidence of structural breaks in the early 2000s by applying the Chow-type-heteroskedasticity-robust Wald-statistic for parameter instability to Granger causality regressions.

The literature on the link between oil price and U.S. exchange rate suggests that the sign of such a link depend on both the source of the shock (oil price shock or exchange rate shock) and the response of the other variable to such a shock.

Looking at the reaction of exchange rate to changes in oil price, there are two strands of the literature. The first strand establishes a negative relationship between oil price and U.S. exchange rate on the basis of the transmission mechanisms through which oil price can be transmitted to the exchange rate. These mechanisms include both the wealth (e.g., [21]) and the terms of trade channels (e.g., [22]). On the one hand, an increase in oil price reduces the USD reserves in oil-importing countries and generates current account imbalances and portfolio reallocation [23]. Consequently, it is expected a depreciation of the domestic currency. On the other hand, a rise in oil price increases import prices in relation to export prices in oil-importing countries, which causes a negative impact on the terms of trade and the depreciation of the domestic currency. The second strand based on petrodollar recycling argument highlights a positive relationship between oil price and U.S. exchange rate. Specifically, after an increase in oil price, oil exporting countries (e.g., OPEC members) increase their demand for assets nominated in USD, which pushes up the USD exchange rate [21].

Looking at how oil price reacts to changes in the U.S. exchange rate, the related literature shows a negative link between oil price and U.S. exchange rate. On the one hand, oil price changes due to an increase in the attractiveness of oil and other commodities as a form of alternative asset against the fall in the price of U.S. assets and USD depreciation (the so-called financialization of the commodity markets or portfolio rebalancing argument; see, e.g., [15, 24]). On the other hand, oil price changes due to movements in world oil markets. On the basis of the law of one price for tradable goods, authors such as [25] argue that given that crude oil is an international commodity traded in USD, an appreciation of the USD increases oil price measured in terms of the domestic currency, which reduces oil demand and, consequently, oil price declines.

Additionally, the related literature also shows how the indirect channels may have an impact on the relationship between oil price and U.S. exchange rate. Thus, [15] highlight that the U.S. restrictive monetary policy may give rise to a USD appreciation due to higher interest rates and a decline in oil price due to lower oil demand.

Therefore, the devaluation of the USD may increase world oil price (negative relationship) for two reasons: i) a rise in oil demand in oil-importing countries and a decline in oil supply in oil-exporting countries (world oil market movements); and ii) a lower return on the USD denominated financial assets and, consequently, an increase in the attractiveness of oil and other commodities as alternative assets (portfolio rebalancing argument). On the other hand, following a rise in oil price, there is a depreciation in the U.S. exchange rate (negative relationship) due to terms of trade and wealth effects, but an appreciation (positive relationship) originated by petrodollar recycling argument. Finally, there could be external shocks like U.S. interest rate increases, which lead to a USD appreciation and a decline in oil price.

## 3 Data

### 3.1 Data description

We consider monthly data for the nominal oil price, which is defined as the spot price of West Texas Intermediate in USD per barrel and is taken from Federal Reserve Economic Data (FRED) (https://fred.stlouisfed.org), and the U.S. nominal narrow effective exchange rate published by the Bank for International Settlements (http://www.bis.org). As [26] defined, a nominal narrow EER is an index based on a trade-weighted average of bilateral exchange rates from a narrow group of trading partners and calculated as geometric weighted averages of such exchange rates, with the weights being based on manufacturing trade flows and capturing both direct bilateral trade and third-market competition by double-weighting following the idea of [27]. The Bank for International Settlements reports the narrow EER comprising 27 economies and the weighting matrix for each period of time is given at web page http://www.bis.org. It is also worth noting that [26] states “EERs provide a useful summary indicator of the overall strength or weakness of a country’s currency. EERs can thus serve various purposes: as a measure of international price and cost competitiveness, as components of monetary/financial conditions indices, as a gauge of the transmission of external shocks, as an intermediate target for monetary policy or as an operational target.” The sample period runs from January 1974 to July 2019, with a total number of 547 observations. The floating exchange rate period starts in 1973, we consider data from 1974 onwards in order to avoid the possible turbulences of the 1973 transition year. We do not consider data before 1973 due to the existence of “Bretton Woods” fixed exchange rate.

### 3.2 Identifying shock episodes

We study the relationship between oil price and U.S. effective exchange rate with a special focus on shock episodes occurred for both variables. The identification of these shocks is innocuous for the econometric analysis performed. Such identification only allows us to show the reaction of one variable to the changes in the other with credible intervals in specific shock episodes.

Similarly to [28], we define a shock episode as a period that involves a cumulative change larger than 40% (positive or negative) in the log of the variable of interest. Thus, we identify ten oil price shock episodes, of which 6 are positive and 4 are negative. Table 1 shows the timing and duration of shock episodes together with the main event occurred in each period. Moreover, most of the shocks characterized by larger duration and high growth rates have occurred since 2000. In fact, the longest and sharpest oil price shock episode started in December 2001. Additionally, we also identify three shock episodes related to exchange rate movements.

**Table 1:** Timing and duration of shock episodes.

	Start date	End date	Months	Total growth (%)	Main event
Oil price shocks					
1	Jan-79	Apr-80	16	98	Iran revolution
2	Nov-85	Mar-86	5	-89	OPEC collapse
3	Jun-90	Oct-90	5	76	Gulf war
4	Dec-96	Dec-98	25	-81	Asian crisis
5	Jan-99	Nov-00	24	105	OPEC cutbacks
6	Dec-01	Jun-08	79	194	Asian boom
7	Jul-08	Dec-08	6	-118	Great crisis
8	Jan-09	Apr-11	28	103	Gradual global recover
9	Jun-14	Jan-16	20	-125	Weak growth
10	Mar-16	Jul-18	29	85	Global growth
U.S. EER shocks					
1	Oct-78	Mar-85	78	42	U.S. monetary policy
2	Apr-85	Apr-88	37	-45	Plaza Accord
3	Feb-02	Jul-08	78	-42	U.S. monetary policy

### 3.3 Granger causality

**Fig 4:**
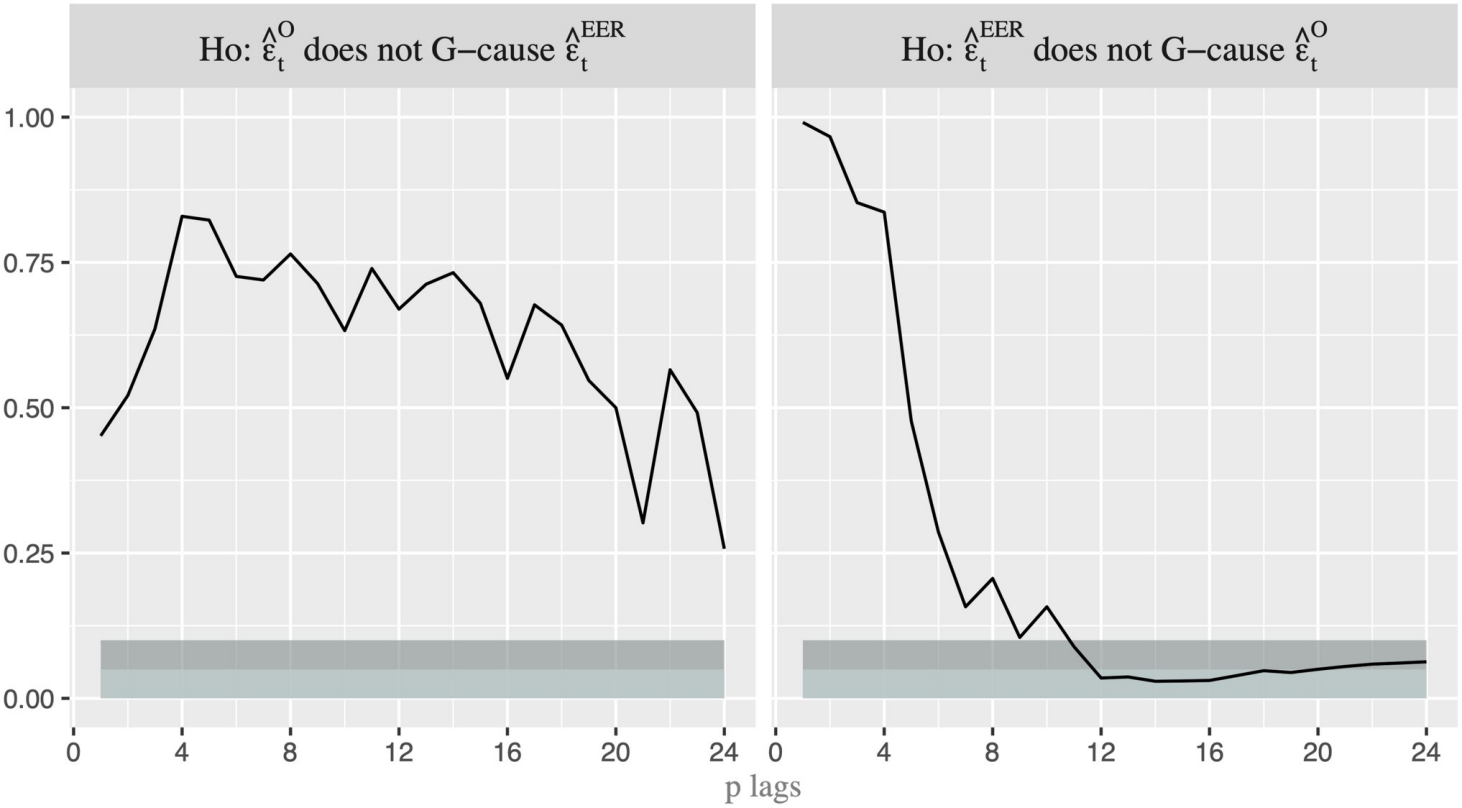
*p-* values for the nonlinear Granger-causality test. p-value for the nonlinear G-causality test with lags p = 1,…,24. Light shaded area represents the rejection of the null hypothesis at the 5% critical level, while dark shaded area represents the rejection at the 10% level.

As a first step, we analyze which variable anticipates the movements of the other in order to establish the order in which the variables should enter into the recursive model. In doing so, we first apply the linear Granger (G) causality test between nominal oil price (*O*_*t*_) and U.S. EER (*EER*_*t*_). Fig 3 shows that the null hypothesis that exchange rate does not G-cause oil price is only rejected at the 10% critical level when *p* = 1, while the reverse G-causality (from oil price to exchange rate) is never rejected. However, we are conscious that the linear test might well not capture properly the true relationship since the linear causality is not able to identify nonlinear linkage mechanism. Thus, we apply the nonlinear G-causality test proposed by [29] -henceforth DP- to the de-linearized series obtained by using the VAR filter, whose number of lags are 2 based on Schwarz Information Criterion. It is worth noting that “by removing linear predictive power with a linear VAR model, any remaining incremental predictive power of one residual series for another can be considered nonlinear predictive power” (see [30], page 1648). Fig 4 shows the *p*-*values* of the DP test for 24 lags. While we cannot reject the null hypothesis that the residuals of oil price equation ${\hat{\varepsilon }}_{t}^{O}$ do not G-cause the residuals of EER equation ${\hat{\varepsilon }}_{t}^{EER}$, we can do it when the reverse causality is considered and several lags are included. Therefore, it seems that the G-causality runs from exchange rate to oil price. This allows us to consider the order [*EER*_*t*_, *O*_*t*_] when we apply VAR models. In other words, we use a recursive identification strategy, in which exchange rate reacts to shocks in oil price only with a delay while oil price responds also contemporaneously to changes in exchange rate.

## 4 Methodology

### 4.1 Previous considerations

Before studying the relationship between oil price and U.S. effective exchange rate, we investigate the stochastic properties of both time series. The results for both augmented Dickey-Fuller test (which does not consider the presence of structural breaks) and [31] test (which takes into account the existence of structural breaks)(both available upon request) indicate that U.S. EER and oil price seem to be *I*(1). The [31] test has been performed after finding the presence of structural breaks by means of the use of the methodology proposed by [32, 33] and specified to analyze endogenous changes in a univariate time series. In addition, given that both variables seem to be *I*(1), we test for cointegration by using the Johansen cointegration test (which only detects stable linear relationships), the [34] test (which allows the existence of unknown endogenous structural breaks) and the [35] test (which detects nonlinear relationships). The results of these cointegration tests (available upon request) indicate the lack of cointegration between the two variables (we would like to thank Daiki Maki and Joerg Breitung for graciously sharing their GAUSS codes). In any case, some authors [36–38] state that it is possible to perform the study in levels allowing for implicit cointegrating relationships in the data if there are and still have consistent estimates of the parameters. Additionally, there have been several authors in the macroeconomic literature that consider the levels/log-levels of *I*(1) variables in the VAR models (see, e.g., [39–41], among others). Finally, it has to be indicated that both including misspecified cointegrating relationship in the VAR model in levels would give rise to biased estimates and estimating the VAR model in the first differences would lead to a loss of information in the levels [36].

### 4.2 Time-invariant VAR model

We analyze the relationship between oil price and U.S. effective exchange rate by using a recursively identified bivariate TVP-VAR model. However, we first estimate a recursively identified bivariate (time-invariant) VAR model with U.S. EER and the nominal oil price as variables (entering into the model in that order) in order to provide a comparative perspective. Unlike this paper, there is a strand of the literature on the effects of oil shocks that considers Bayesian methods to time-invariant VAR models (see, e.g., [42–45], among others).

The reduced form is written as

$$\begin{array}{c} y_{t}=a+\sum_{j=1}^{p}\Phi \left(p\right)y_{t-j}+u_{t} \end{array}$$ (1)

with *y*_*t*_ being a (2 × 1) vector that contains the U.S. EER and the nominal oil price, and with *u*_*t*_ being a generalization of a white noise process with variance-covariance matrix Ψ.

We select two lags (*p* = 2) based on Schwarz Information Criterion. This choice is consistent with other studies of the related literature (see [1]; [18]). To identify the bivariate VAR model, we consider the ordering in which exchange rate has a contemporaneous effect on oil price, but not the reverse. This ordering is based on the results of causality described in the previous Section. We calculate the impulse response of exchange rate to one unit oil price shock, the impulse response of oil price to one unit exchange rate shock and their one standard deviation confidence bands obtained through bootstrap procedure. In particular, we apply the Efron bootstrap percentile confidence interval with 10,000 draws.

### 4.3 TVP-VAR model

It seems clear that the relationship between nominal oil price and U.S. EER has changed over time (see Figs 1 and 2). Thus, we consider a time-varying parameter (TVP) model similar to the model implemented in [46] and [11]. In particular, we consider a recursively identified bivariate TVP-VAR model with U.S. EER and the nominal oil price as variables (entering into the model in that order). This model allows us to capture the effects of oil price (exchange rate) shocks over time by means of a flexible approach, with the VAR coefficients and variance-covariance matrix changing over time.

The following TVP-VAR model is considered:

$$\begin{array}{c} y_{t}=a_{t}+\sum_{j=1}^{p}A_{j,t}\;y_{t-j}+\mu_{t} \end{array}$$ (2)

where *y*_*t*_ is a (2 × 1) vector that contains exchange rate and oil price with *t* = 1, …, *T*; *a*_*t*_ is a (2 × 1) vector of time-varying (TV) coefficients that multiply constant terms; *A*_1,*t*_, …, *A*_*p*,*t*_ are (2 × 2) matrices of TV coefficients, and *μ*_*t*_ is a (2 × 1) vector of heteroskedastic unobservable shocks with (2 × 2) variance-covariance matrix *Ω*_*t*_ (specifically, $\mu_{t}∼N\left(0,\Omega_{t}\right)$).

This model can be rewritten as:

$$\begin{array}{c} y_{t}=\left(I_{2}⊗X_{t}\right)\alpha_{t}+\mu_{t} \end{array}$$

where *I*_2_ is a 2-dimensional identity matrix; ⊗ denotes the Kronecker product; $X_{t}=\left[1,y_{t-1}^{\prime },\ldots y_{t-p}^{\prime }\right]$ is the vector of 1 × (1 + 2*p*) explanatory variables, and *α*_*t*_ = *vec*(*A*_*t*_) is the stacked vector of TV coefficients *A*_*t*_ = (*a*_*t*_ *A*_1,*t*_…*A*_*p*,*t*_).

Following [46], we consider the triangular reduction of the variance-covariance matrix Ω_*t*_:

$$\begin{array}{c} B_{t}\Omega_{t}B_{t}^{\prime }=\Sigma_{t}\Sigma_{t}^{\prime } \end{array}$$

where *B*_*t*_ is the lower triangular matrix of error covariances with ones on the diagonal and Σ_*t*_ is the diagonal matrix with diagonal elements being the TV error deviations. This decomposition of the variance-covariance matrix mitigates the proliferation of parameters problems, which is important in this TVP-VAR. Although the order of the variables could matter given the lower matrix *B*_*t*_, the results with TV covariances are very similar with the reverse order. In our case,

$$\begin{array}{c} B_{t}=[\begin{array}{cc} 1 & 0\\ b_{21,t} & 1 \end{array}]\;\;\Sigma_{t}=[\begin{array}{cc} \sigma_{1,t} & 0\\ 0 & \sigma_{2,t} \end{array}] \end{array}$$

Therefore, the TVP-VAR model is written as:

$$\begin{array}{c} y_{t}=\left(I_{2}⊗X_{t}\right)\alpha_{t}+B_{t}^{-1}\Sigma_{t}\varepsilon_{t}, \end{array}$$

with $\varepsilon_{t}∼N\left(0,I_{2}\right)$.

The dynamics of the TV parameters is specified as:

$$\begin{array}{c} \alpha_{t}=\alpha_{t-1}+\nu_{t} \end{array}$$

$$\begin{array}{c} b_{t}=b_{t-1}+\zeta_{t} \end{array}$$

$$\begin{array}{c} log\;\sigma_{t}=log\;\sigma_{t-1}+\eta_{t} \end{array}$$

where *α*_*t*_ describes the dynamics of the coefficients, *b*_*t*_ describes the dynamics of the non-zero and non-one elements of matrix *B*_*t*_ and *σ*_*t*_ describes the dynamics of the diagonal matrix Σ_*t*_.

It is assumed that the error terms (*ε*_*t*_, *ν*_*t*_, *ζ*_*t*_, *η*_*t*_) are jointly normally distributed with the variance covariance matrix (V) being:

$$\begin{array}{c} V=Var([\begin{array}{c} \begin{array}{c} \varepsilon_{t}\\ \nu_{t}\\ \zeta_{t}\\ \eta_{t} \end{array} \end{array}])\;=[\begin{array}{cccc} I_{2} & 0 & 0 & 0\\ 0 & Q & 0 & 0\\ 0 & 0 & S & 0\\ 0 & 0 & 0 & W \end{array}] \end{array}$$

with *Q* and *W* being positive definite matrices and *S* is positive. It is worth noting that *S* here is (1 × 1) given there is only one element of matrix *B*_*t*_ different from zero and different from one.

The priors basically follow the same principles as in [46] and are summarized in Table 2. Unlike [46] who fixed *k*_*S*_ = 0.1, we have chosen *k*_*S*_ = 1 because this allows us to better capture the high volatility of both variables in the short-term. As [46] warns, posterior inference may be affected by the choice of *k*_*Q*_, *k*_*S*_ and *k*_*W*_. Thus, as a robustness check (available upon request), we also compute posterior inference using alternative values for *k*_*Q*_, *k*_*S*_ and *k*_*W*_. Specifically, following [46], we consider all possible combinations of *k*_*Q*_ = {0.01; 0.05; 0.1}, *k*_*S*_ = {0.01; 0.1; 1} and *k*_*W*_ = {0.005; 0.01}. The results are highly similar to those of our baseline specification.

**Table 2:** Prior distributions. N and IW denote the normal and independent inverse-Wishart distributions. ${\hat{\alpha }}_{OLS},{\hat{b}}_{OLS}$ and ${\hat{\sigma }}_{OLS}$ are the OLS estimates in a time-invariant VAR model obtained from the training sample. $V({\hat{\alpha }}_{OLS})$ is the corresponding estimator of the covariance matrix of ${\hat{\alpha }}_{OLS}$ and $V({\hat{b}}_{OLS})$ is the estimated variance of ${\hat{b}}_{OLS}$.

Parameters		
	Prior family	Coefficients
*α*_0_	$N({\hat{\alpha }}_{OLS}\;,\;k_{\alpha }\times V\left({\hat{\alpha }}_{OLS}\right))$	*k*_*α*_ = 4
*b*_0_	$N({\hat{b}}_{OLS}\;,\;k_{b}\times V\left({\hat{b}}_{OLS}\right))$	*k*_*b*_ = 4
log *σ*_0_	N(log ${\hat{\sigma }}_{OLS}\;,\;k_{\sigma }\times I_{2}$)	*k*_*σ*_ = 1
Hyperparameters		
	Prior family	Coefficients
*Q*	$IW(k_{Q}^{2}\times pQ\times V\left({\hat{\alpha }}_{OLS}\right)\;,\;pQ)$	*k*_*Q*_ = 0.01
		*pQ* = 60
*S*	$IW(k_{S}^{2}\times pS\times V\left({\hat{b}}_{OLS}\right)\;,\;pS)$	*k*_*S*_ = 1
		*pS* = 2
*W*	$IW(k_{W}^{2}\times pW\times I_{2}\;,\;pW)$	*k*_*W*_ = 0.01
		*pW* = 3

These prior distributions are used to carry out Bayesian inference that involves Markov Chain Monte Carlo (MCMC) posterior simulation methods (Gibbs sampler) for the unobservable states *α*^*T*^, *B*^*T*^, Σ^*T*^ (referred to the entire path of parameters ${\left\{\alpha_{t}\right\}}_{t=1}^{T},{\left\{B_{t}\right\}}_{t=1}^{T}$ and ${\left\{\Sigma_{t}\right\}}_{t=1}^{T}$, respectively.) and the hyperparameters of the variance-covariance matrix *V*. The MCMC algorithm implemented is the algorithm 3 proposed by [11].

The simulations are based on 50,000 iterations for the Gibbs sampler, with a burn-in of 5,000. The length of the training sample used for determining prior parameters via least squares is 60 (i.e., the first 5 years of the sample, 1974:01-1978:12) and the lag length used is 2. Therefore, the first date for which time-varying standard deviations of the residuals and time-varying responses are obtained is 1979:03.

## 5 Results

### 5.1 (Time-invariant) VAR model

**Fig 5:**
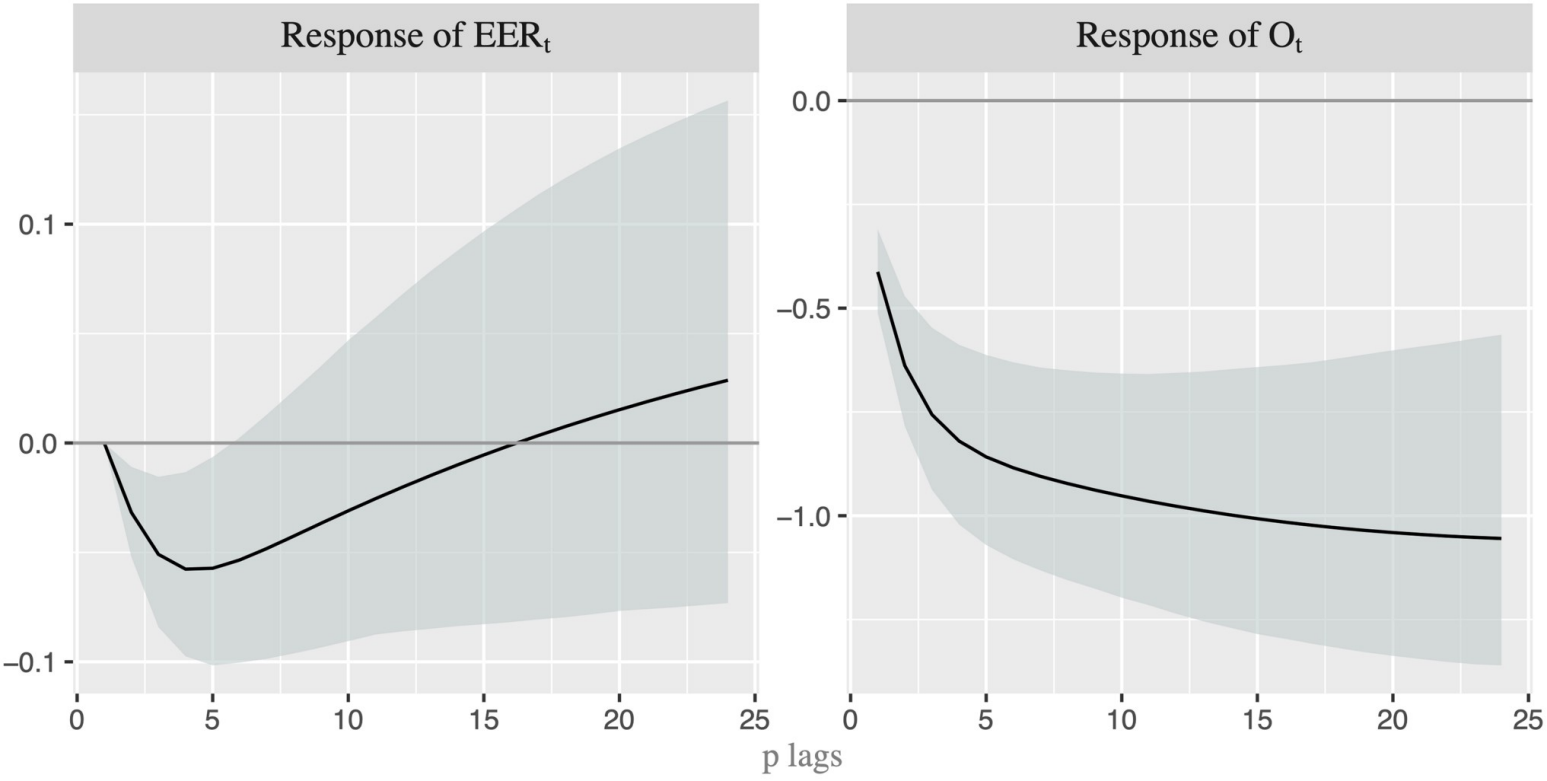
Responses of U.S. EER and oil price to one unit shock of the other variable in the (time-invariant) VAR model (1974:01-2019:07). Shaded intervals represent one standard deviation confidence bands obtained by bootstrapping.

The impulse response functions from the (time-invariant) VAR model are depicted in Fig 5. On the one hand, they show that an oil price increase leads to a depreciation of the U.S. EER during the first months after the shock and an appreciation afterwards. Thereby, while the short-run reaction (i.e., the depreciation) is statistically significant, the long-run response (i.e., the appreciation) is not significant. On the other hand, the impact of a positive shock on U.S. EER tends to significantly reduce oil price. Thus, the responses in favor of a negative relation between oil price and exchange rate are statistically significant, while those that imply a positive relation are not statistically significant. Therefore, these findings show evidence in favor of a negative relationship between oil price and exchange rate, which is in concordance with most of the economic theory previously highlighted in the Introduction.

### 5.2 TVP-VAR model

**Fig 6:**
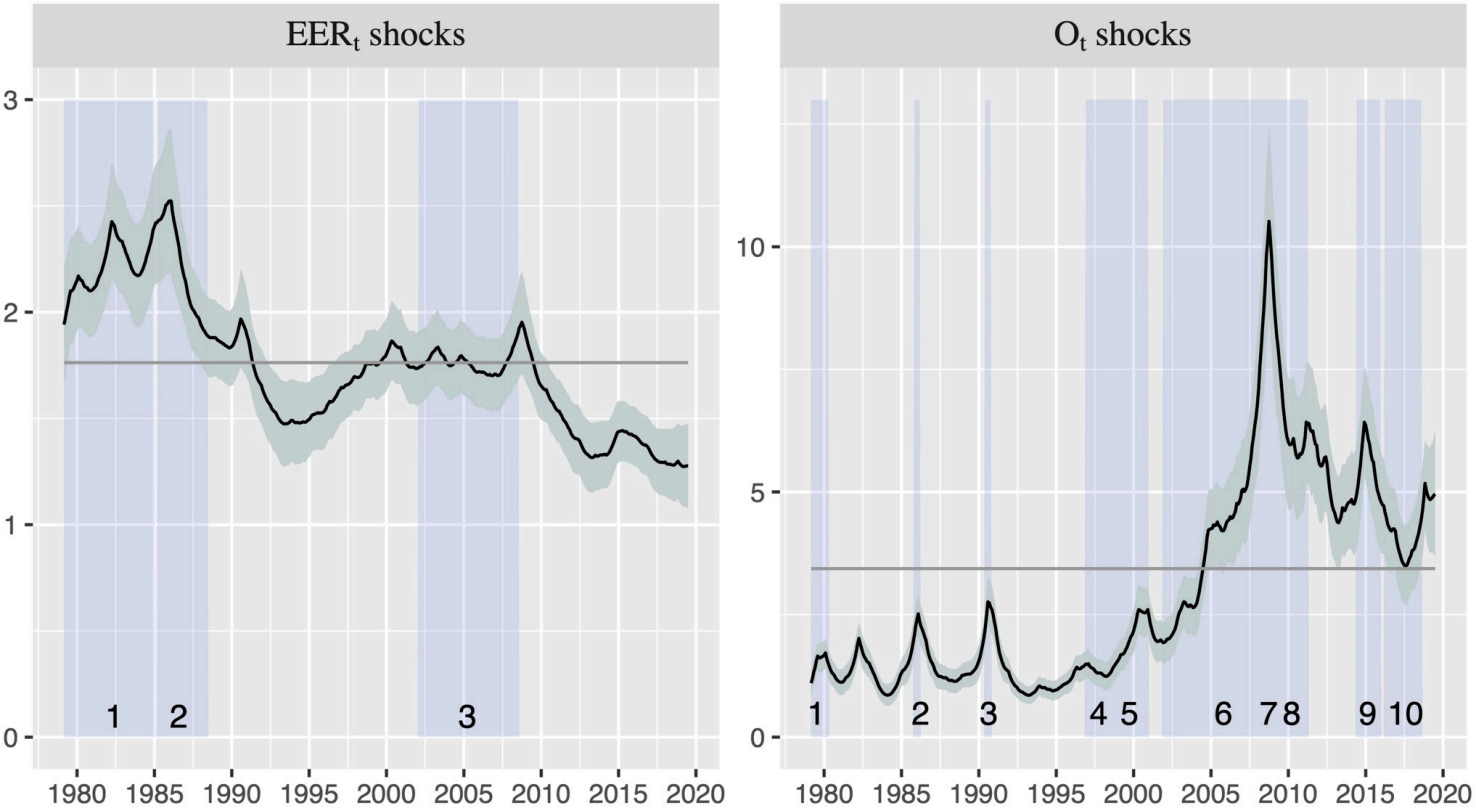
Time-varying standard deviations of the residuals of the U.S. EER and the oil price equations (1979:03-2019:07). The horizontal gray line shows the standard deviations in the (time − invariant) VAR model. The black line displays the mean of the standard deviations while the grey area refers to the 16th and 84th percentiles. Vertical shaded areas correspond to the episodes depicted in Table 1.

Unlike the (time-invariant) VAR model where the standard deviations of the error terms for U.S. EER and oil price are constant (with the estimation being 1.762 and 3.437, respectively), the TVP-VAR model allows the standard deviations change over time. Thus, Fig 6 shows the TV standard deviations obtained from the estimation of the TVP-VAR model, which represent the shocks (unexpected movements) on U.S. EER and oil price that would have been influenced by other turmoil in, for example, global or financial markets. The vertical shaded areas in Fig 6 correspond to the different shock episodes for U.S. EER and oil price depicted in Table 1. This Figure shows that exchange rate shocks were comparatively large before 1994 relative to the size of the shocks from that date onwards, while the opposite occurs with oil price shocks (which become increasingly high after 2000, with a peak in 2008). Interestingly, there is a striking coincidence between the historical U.S. EER and oil price shock episodes and the peaks in the estimated volatility, which suggests that the estimated shocks derived from the TVP-VAR model seem to provide a more realistic approach than the one obtained from the standard VAR model. This finding reinforces the evidence showed in Fig 2, suggesting that the dynamic relationship between both variables has been strongly influenced by the volatility related with economic and political events.

Following the referee suggestion, the three-dimensional (3D) plot of time varying responses of one variable to one unit shock of the other variable is not included in the main text and has been relegated to an appendix available upon request. Notice that the 3D plots are generally not easy to read and interpret.

**Fig 7:**
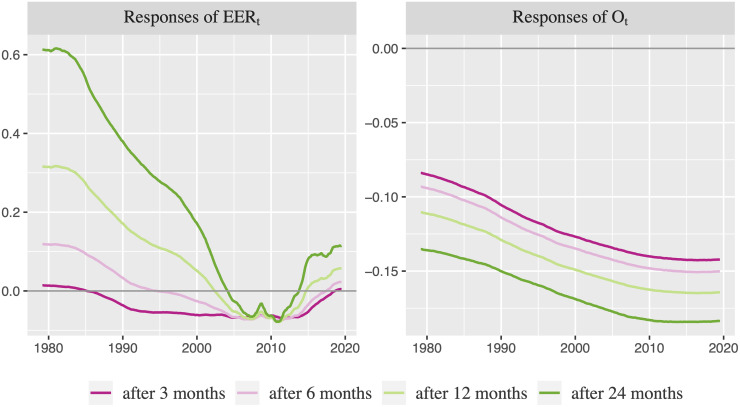
Responses of U.S. EER and oil price to one unit shock of the other variable after 3, 6, 12, 24 months (1979:03-2019:07).

Fig 7 shows the responses after 3 months, 6 months, 12 months and 24 months for each period of time in which the shock happens in order to compare the effects over time. It is observed that an increase in oil price leads to a depreciation of U.S. EER in the short-run (responses after 3 months) during most of the sample period, especially since 1990. [3] and [4] also report a USD depreciation at short term after an increase in oil price. However, the depreciation observed in Fig 7 is only statistically significant between the mid-2000s and the mid-2010s (see Fig A.1 from S1 Appendix). These responses turn out positive (i.e., an appreciation appears) in the long-run for the whole period, with the exception of the period between 2002 and 2014, in which the depreciation remains (not being statistically significant). Moreover, the long-run responses seem to be stronger before the beginning of the 2000s than later on, with the appreciation before the 1990s being statistically significant. Therefore, the results show evidence in favor of the heterogeneity in the response although there is a similar sign pattern over time: U.S. EER reacts negatively to oil price increases in the short-run and positively in the long-run before the 2000s.

The depreciation of USD (in effective terms) in the short-run after an oil price increase can be explained by: (i) the wealth channel, which implies that wealth (measured in USD) is transferred from oil-importing economics to oil-exporting countries reducing the USD reserve of the former countries and improving current account balance of the latter; and (ii) the terms of trade channel, which implies that a rise in import prices in relation to export prices in oil-importing countries leading to a negative effect on terms of trade. The appreciation of USD (in effective terms) in the long-run after a rise in the oil price can be explained by the petrodollar recycling argument, which indicates that oil-exporting economics increase their purchase preferences for assets nominated in USD. Additionally, the responses of U.S. EER to oil price increases may be also related to the strong effects of the U.S. monetary policy carried out by the Federal Reserve on exchange rate during specific periods of time.

The statistically significant appreciation of USD (in effective terms) observed in the long run before 1990s may be explained by a combination of the effects of petrodollar recycling and the effects on exchange rate due to the U.S. restrictive monetary policy carried out in the 1980s to fight against inflation. However, the statistically significant depreciation observed in the short-run between the mid-2000s and the mid-2010s may be explained through the wealth channel and the trade balance adjustment, with the role of petrodollar recycling and the monetary policy reaction being negligible. Notice that the U.S. expansionary monetary policy performed by the Federal Reserve just after the subprime mortgage crisis may have also influenced in the significance of the depreciation in the short-run during the beginning of the Great crisis.

**Fig 8:**
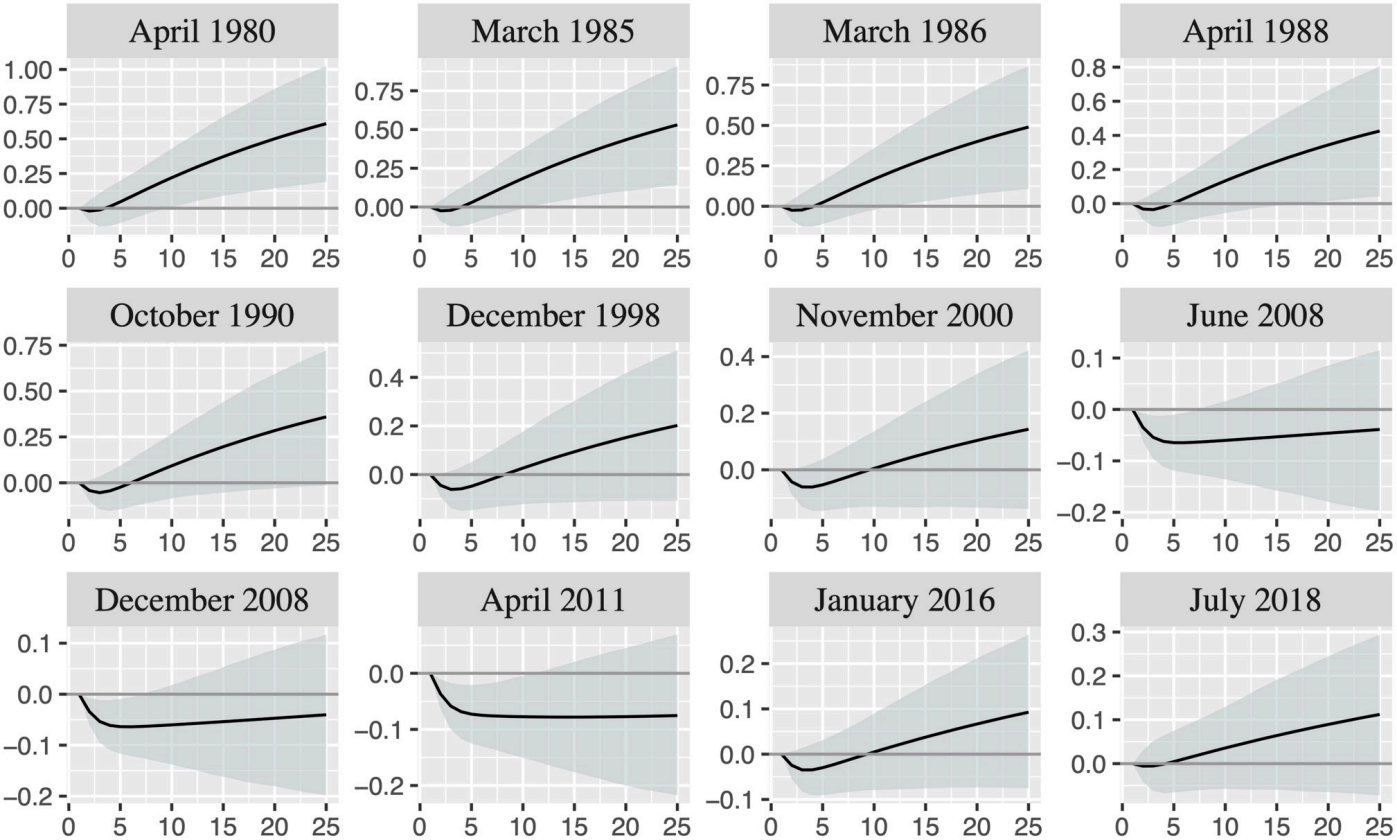
Responses of U.S. EER to one unit oil price shock in the shock episodes depicted in Table 1. The black line displays the mean while the grey area represents the 16th and 84th percentiles.

To analyze the extent to which the reactions of U.S. EER to oil price shocks are similar across different shock episodes, Fig 8 presents the responses of U.S. EER to one unit oil price shock together with the 16th and 84th percentiles for the ten oil price shock episodes and the first two exchange rate shock episodes depicted in Table 1. The seventh oil price shock episode and the third exchange rate shock episode end in June 2008 and July 2008, respectively. Thus, we only present the responses for the end of the oil price shock episode to save space. Moreover, we only present the responses in the date that corresponds to the end of the shock episodes. The responses are broadly similar for any date we consider inside a specific shock episode.

Fig 8 shows that the responses are negative in the short-run and turn out positive in the long-run in most episodes. However, the oil price movements have only had a significant statistically influence on exchange rate in the long-run in the shock episodes occurred before the 1990s and in the short-run in the shock episodes occurred between the mid-2000s and the mid-2010s.

**Fig 9:**
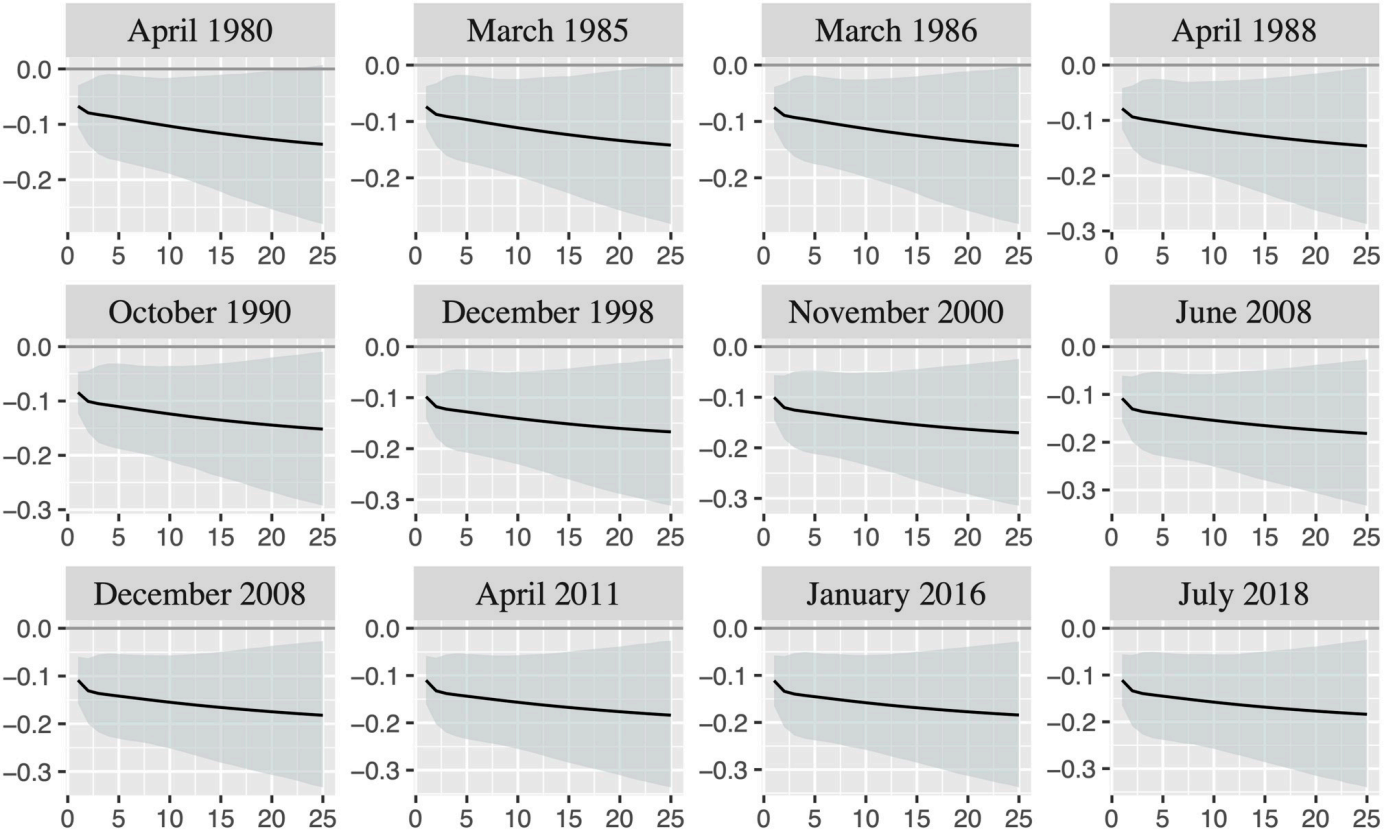
Responses of oil price to one unit U.S. EER shock in the shock episodes depicted in Table 1. The black line displays the mean while the grey area represents the 16th and 84th percentiles.

Whereas the sign of the responses of U.S. EER to an oil price shock changes over time, the responses of oil price to an appreciation of U.S. EER is always negative (see Fig 7). In particular, the responses are negative in the short- and long-run (negative reactions at short-run are also found by [3]), although the reactions are more intensive from the beginning of the 1990s onwards. The reactions differ across time periods with all being statistically significant (see Fig A.2 from S1 Appendix) and with the negative reaction being higher over time. These responses can be better appreciated when we look at the responses across different shock episodes (Fig 9). Thus, Fig 9 shows that the responses are negative and statistically significant for the oil price shock and exchange rate shock episodes. Therefore, oil price declines after an appreciation of U.S. EER, which is in concordance with the economic theory. In particular, an appreciation of U.S. EER makes USD stronger with respect to currencies of its main trading partners. Thus, purchases in international markets in USD are more expensive for countries different from the U.S. after an appreciation of U.S. EER, which reduce the world demand of oil and other international commodities traded in USD and, consequently, also reduce the oil price. Likewise, the value gain of USD with respect to foreign currencies reduces the attractiveness of oil and other commodities as alternative assets, which reduces the oil demand and its price. That is, the argument of financialization of the commodity markets works the other way around.

## 6 Conclusions

Conventional wisdom holds that exchange rate is an important determinant of the trade balance, which is one of the key components of GDP, so that any movement in the exchange rate can have a relevant effect on the evolution of macroeconomic variables. Moreover, the USD exchange rate is especially relevant because USD is the standard currency of international trade. Besides, crude oil, which is considered to be a basic input to production, one of the main representatives of the large commodity markets and so one of the main indicators of economic activity worldwide, is priced in USD (e.g., [12, 47–49]). Therefore, the evolution of the exchange rate and crude oil markets is closely related and there is no doubt about the interest of knowing the relationship between both markets for decision makers, since their movements can influence key macroeconomic indicators.

This paper analyzes the dynamic interactions between U.S. exchange rate and oil price by considering a TVP-VAR model with the use of monthly data from 1974 to 2019, so filling out the gap existing in the scarce literature on this issue [3, 4]. Unlike the (time-invariant) VAR model which considers that the responses of one variable to the shock of the other are equal across different periods of time, the TVP-VAR model allows that these responses change over time without establishing specific breaks, which allows us to capture the evolution of the reactions over time without depending on subsamples. Moreover, unlike [3], this paper studies the reactions of each variable to a shock in the other for horizons different from one-period-ahead for the whole sample period (not only for specific dates), includes credible intervals and analyzes how responses are in the period before the 2000s (period in which half of the oil price and exchange rate shocks have occurred). Unlike [4], this paper also analyzes the response of oil price to shocks in the exchange rate, incorporates credible intervals and studies what has happened in the period before the mid-1990s (period in which the most well-known oil price shocks have occurred).

The negative sign patterns of U.S. EER to oil price shocks in the short-run are in line with the results of [3] and [4] and highly similar across different time periods, although the responses are only statistically significant between the mid-2000s and the mid-2010s. The depreciation in the short-run after an oil price shock is consistent with the transmission through the wealth and the terms of trade channels. In other words, our findings suggest that the oil price increase observed since the 2000s has led to a wealth transfer from the U.S. to oil-exporting countries and has forced a trade balance adjustment, giving rise to the depreciation of the U.S. EER. In contrast, the long-run responses of U.S. EER to oil price shocks show qualitative and quantitative differences. These responses are positive before the mid-2000s, but negative reactions appear afterwards although they are only statistically significant before the 1990s. The finding that appreciation is statistically significant before the 1990s is in concordance with both the petrodollar recycling argument and the reactions of exchange rate to the aggressive monetary policy performed to control inflation during the Volcker regime.

The depreciation of U.S. EER after an oil price shock may have positive effects in oil-importing countries with currencies different from the USD since the exchange rate may absorb partially the impact of such a shock and so may dampen the effects of sharp changes in oil price.

The oil price significantly declines after an appreciation of U.S. EER during the whole sample, which is in conformity with previous studies (e.g., [3]), as well as with the theoretical arguments about the decline of oil price through lower oil world oil demand and/or reduction in the attractiveness of oil as a class of alternative asset. The short- and long-run negative patterns of responses are similar, with the reactions being more intensive over time. However, no remarkable specific reactions are found in the periods of economic turmoils such as the global financial crisis.

An appreciation of U.S. EER makes oil more expensive in local currency for “non-USD” economies, which leads to a decline in oil consumption and, as a consequence, the oil price is reduced due to the reduction in oil demand.

Looking at the responses in the shock episodes, we observe the same pattern previously described. In particular, the reaction of U.S. EER to an oil price shock is negative in the short-run and positive in the long-run for most episodes, but it is only statistically significant for the shock episodes before the 1990s and for the shock episodes between the mid-2000s and the mid-2010s. The responses of oil price to an appreciation of U.S. EER are negative and statistically significant, being more intensive after the 1990s.

Therefore, these findings highlight the importance of considering the period of time in which the oil price shock occurs because the U.S. EER response may differ over time and, consequently, the economic policy reaction which is required to counteract such a shock may also differ. Moreover, the decline in oil price observed after an appreciation of U.S. EER is not the same over time and it may generate different adverse effects on investment depending on the period of time the appreciation takes place. The knowledge of such effects may help financial investors to diversify their investments in order to optimize the risk-return profile of their portfolios.

In future work, it would be interesting to analyze the time-varying effects of oil price shocks on U.S. EER depending on their underlying source (oil supply shocks, aggregate demand shocks and oil specific-demand shock).

## Supporting information

S1 Appendix (PDF)

S1 Data (RAR)
