# Supplementary material for: Dynamic interactions between oil price and exchange rate
Source: PLoS One. 2020 Aug 20;15(8):e0237172. doi: 10.1371/journal.pone.0237172 (PMC7444559; doi:10.1371/journal.pone.0237172)
Supplement: S1 Appendix — (PDF) [file pone.0237172.s001.pdf]

Appendix A

This Appendix presents the responses of one variable to one unit shock in the other after 3 months, 6 months, 12 months and 24 months for each period of time in which the shock happens, as well as their credible intervals.

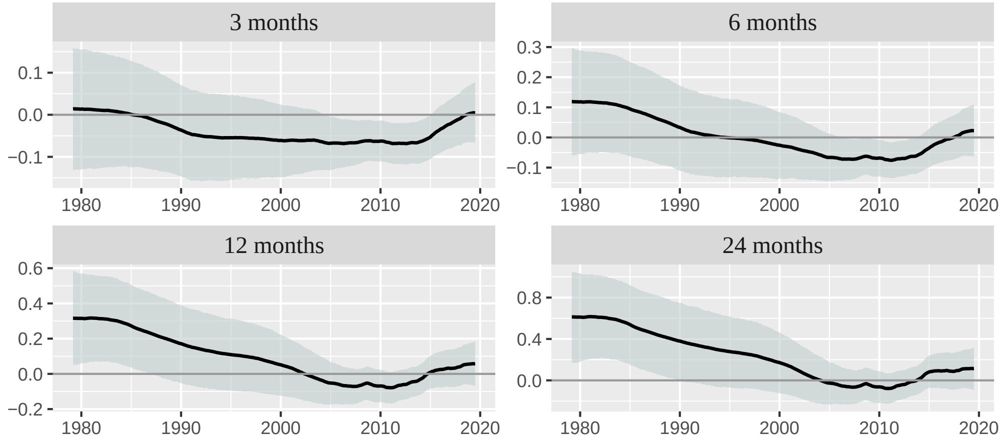

Note: The black line displays the mean while the grey area represents the 16th and 84th percentiles.

Fig A.1. Responses of U.S. EER to one unit shock of oil price after 3, 6, 12 and 24 months (1979:03-2019:07)

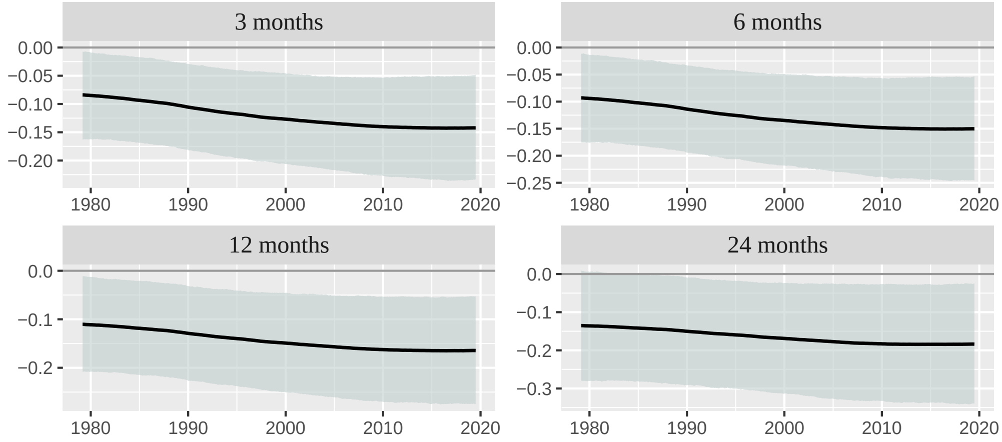

Note: The black line displays the mean while the grey area represents the 16th and 84th percentiles

Fig A.2. Responses of oil price to one unit U.S. EER shock after 3, 6, 12 and 24 months (1979:03-2019:07)
